# An exploratory university-based cross-sectional study of the prevalence and reporting of mistreatment and student-related factors among Thai medical students

**DOI:** 10.1186/s12909-023-04462-3

**Published:** 2023-06-26

**Authors:** Waravudh Naothavorn, Pongtong Puranitee, Winitra Kaewpila, Sutida Sumrithe, Sylvia Heeneman, Walther N. K. A. van Mook, Jamiu O. Busari

**Affiliations:** 1 Chakri Naruebodindra Medical Institute, Faculty of Medicine Ramathibodi Hospital, Mahidol University, Samut Prakan, Thailand; 2 Department of Pediatrics, Faculty of Medicine Ramathibodi Hospital, Mahidol University, Bangkok, Thailand; 3 Department of Pathology, School of Health Professions Education, Faculty of Health, Medicine & Life Sciences (FHML), Maastricht University (UM), Maastricht, The Netherlands; 4 Department of Intensive Care, and Academy for Postgraduate Medical Training, Maastricht University Medical Centre+, and School of Health Professions Education, Faculty of Health, Medicine & Life Sciences (FHML), Maastricht University (UM), Maastricht, The Netherlands; 5 Department of Educational Development & Research, Faculty of Health, Medicine & Life Sciences (FHML), Maastricht University (UM), Maastricht, The Netherlands and Department of Pediatrics and HOH Academy, Horacio Oduber Hospital, Oranjestad, Aruba

**Keywords:** Burnout, Medical education, Mistreatment

## Abstract

**Background:**

Mistreatment is a behavior that reflects disrespect for the dignity of others. Mistreatment can be intentional or unintentional, and can interfere with the process of learning and perceived well-being. This study explored the prevalence and characteristics of mistreatment, mistreatment reporting, student-related factors, and consequences among medical students in Thai context.

**Methods:**

We first developed a Thai version of the Clinical Workplace Learning Negative Acts Questionnaire-Revised (NAQ-R) using a forward-back translation process with quality analysis. The design was a cross-sectional survey study, using the Thai Clinical Workplace Learning NAQ-R, Thai Maslach Burnout Inventory-Student Survey, Thai Patient Health Questionnaire (to assess depression risk), demographic information, mistreatment characteristics, mistreatment reports, related factors, and consequences. Descriptive and correlational analyses using multivariate analysis of variance were conducted.

**Results:**

In total, 681 medical students (52.4% female, 54.6% in the clinical years) completed the surveys (79.1% response rate). The reliability of the Thai Clinical Workplace Learning NAQ-R was high (Cronbach’s alpha 0.922), with a high degree of agreement (83.9%). Most participants (*n* = 510, 74.5%) reported that they had experienced mistreatment. The most common type of mistreatment was workplace learning-related bullying (67.7%), and the most common source was attending staff or teachers (31.6%). People who mistreated preclinical medical students were most often senior students or peers (25.9%). People who mistreated clinical students were most commonly attending staff (57.5%). Only 56 students (8.2%) reported these instances of mistreatment to others. Students’ academic year was significantly related to workplace learning-related bullying (*r* = 0.261, *p* < 0.001). Depression and burnout risk were significantly associated with person-related bullying (depression: *r* = 0.20, *p* < 0.001, burnout: *r* = 0.20, *p* = 0.012). Students who experienced person-related bullying were more often the subject of filed unprofessional behavior reports, concerning conflict or arguments with colleagues, being absent from class or work without reasonable cause, and mistreatment of others.

**Conclusions:**

Mistreatment of medical students was evident in medical school and was related to the risk for depression and burnout, as well as the risk of unprofessional behavior.

**Trial registration:**

TCTR20230107006(07/01/2023).

## Introduction

Mistreatment of medical students was initially documented, brought to light, recorded in 1982 [1], through manners such as public humiliation, taking credit for others' work, being threatened with lower grades [2], being systematically denigrated and stigmatized by hostile and aggressive acts [3]. The Association of American Medical Colleges (AAMC) described mistreatment among medical students as an intentional or unintentional behavior that reflects disrespect for the dignity of others and unreasonably interferes with the process of learning [4].

Mistreatment in medical schools is common worldwide. In 1991, the AAMC added a question on mistreatment to its Annual Medical School Graduation Questionnaire to determine the size of the problem [4]. In 1990, a U.S. study found that 80.6% of senior medical students reported being abused by the end of their senior year [5]; subsequently, 85–86 percent of medical students had experienced at least one instance of mistreatment [7]. The prevalence of harassment and discrimination in undergraduate medical training and clerkships was reported to be 59.6% (*n* = 30 studies, 26,579 medical students) [8]. In other countries, the rate of mistreatment of medical students ranges from 62.5 percent (Pakistan) to 63.4 percent (Thailand) to 68.8 percent (Japan) [9–11]. Most commonly reports were person-related bullying, public humiliation, belittlement and verbal abuse, with similar incidences in Western and non-Western countries [4–12].

According to previous literature, mistreatment was conducted by faculty members, house staff (residents and interns), attending staff, nurses, patients, and other students [4–15]. Various student-related factors have been associated with mistreatment of medical students, including being a senior student, and having psychiatric problems or those with a low grade point average (GPA) and being male. In Egyptian culture, male students were said to report or complain more about mistreatment than female students [4, 11–16]. However, these studies did not report on potential related personal and professional factors, such as unprofessional behavior of students.

In Western countries, mistreatment was associated with burnout, anxiety, depression, alcohol or narcotic misuse, suicide attempts, lack of confidence, avoidance, and resignation from healthcare provider roles [7, 14, 17–19]. Mistreatment also leads to stress which can impair learning and memory in students [17, 20]. Whereas a positive stress level can increase learning [21]. A negative level of stress (too much stress) can cause physical and mental health problems [22], reduce self-esteem [23], and influence academic performance [24]. Negative stress among medical students is linked to anxiety, depression, and suicide [14, 17–19, 24–26]. In Singapore, 30% of abused students expressed fear or shame [15]. A 2017 Thai study found that mistreated students felt unpleasant and burned out without depression [11].

Social, psychological, and physical factors that influence or are influenced by academic activities are incorporated into the learning environment and determine how students perceive support systems, learning autonomy, emotive reactions, and the intrinsic value of education [27]. Faculty, nurses, residents, fellows, and other healthcare staff interact socially, psychologically, and physically with medical students in clinical settings. Faculty members influence medical students' risk of mistreatment [28]. Culture and the learning environment have an influence on mistreatment. Disrespectful conduct and abuse in healthcare settings may be tolerated or accepted in hierarchical hospitals [29]. Medical school's hierarchical culture can lead to mistreatment of students, including self-sacrifice to improve patient results [30]. In order to effectively manage the issue, bystanders must recognize, disclose, and respond to maltreatment. Hierarchy, self-sacrifice, perseverance, and reverence prevented students from reporting mistreatment, as they worried about being considered “troublemakers" and risk harming their careers [31]. A Western study found several barriers to effective reporting systems, including students' perception that medical culture includes mistreatment, incidents being considered not important enough to report, a fear that speaking up might damage student–teacher relationships, reporting processes being too difficult, and empathy for the student that conducted mistreatment [30]. Asian cultures value community, collaboration and unity over individual ones [32, 33]. Thus, Asian and Western medical students may be mistreated differently, be aware of mistreatment, view mistreatment and report, review, and adjudicate mistreatment issues differently.

Mistreatment likely affects both Western and non-Western medical students' well-being. However, no Asian study to date has examined how mistreatment affects learning, competence, wellbeing and/or professional identity/professionalism. Exploring undergraduate medical students' experiences of mistreatment and its relationship with aspects like well-being is crucial for detecting and controlling this issue and designing novel approaches for eradicating mistreatment in Asian medical schools. The objectives of this current study were to explore the prevalence, recurrence, categories of mistreatment, post mistreatment reporting actions and related behavior, and well-being among medical students in a Thai context. We hypothesized that mistreatment is related to burnout, depression, and unprofessional behavior of students.

## Methods

This study used a descriptive cross-sectional design.

### Settings and subjects

Medical students studying at the Faculty of Medicine at Ramathibodi Hospital, Mahidol University between years 1 and 6 (*N* = 1,215) in 2021 were invited to participate in a survey using convenience sampling. The sample size was calculated using a 95% confidence level, a margin of error of 5%, a population proportion of 53.7%, and a population size of 1,215. The minimum sample size was calculated as *n* = 276.

All participants provided informed consent before completing the survey. Participants without consent were excluded. Of the 1,215 medical students invited to participate, 961 (79.1%) responded, but 280 did not give consent to participate. There were no incomplete responses. The number of completed survey was 681, representing a response rate of 63.4%. This means that 681 students completed the study.

### Instruments and data collection

The survey and informed consent form were provided to all participants via Google Forms. The questionnaire comprised three parts; 1) participants’ demographic characteristics, 2) mistreatment prevalence and reporting actions, and 3) student-related factors.

#### 1) Participants’ demographic characteristics

The first part of the questionnaire collected demographic data, including age, gender, current year of study, GPA, and underlying diseases (including psychiatric diseases).

#### 2) Mistreatment prevalence and reporting actions

The Negative Acts Questionnaire–Revised (NAQ-R), was originally used for detecting mistreatment of employees in workplaces, and mistreatment was classified using three categories: workplace learning-related bullying, person-related bullying, and physically intimidating bullying [34]. The NAQ-R was later revised by adding two categories (sexual harassment and ethnic harassment) and tested for psychometric features to expand its applicability to health professional students in clinical workplace environments [3, 17], and subsequently referred to as the Clinical Workplace Learning NAQ-R scale.This scale measures the occurrence and recurrence of mistreatment over the last academic year using five categories (workplace learning-related bullying [WLRB], person-related bullying [PRB], and physically intimidating bullying [PIB], sexual harassment [SH], and ethnic mistreatment [EH]). The reliability estimates for all factors of the NAQ-R scale ranged from 0.79 to 0.94 [3].

A Thai version of the Clinical Workplace Learning NAQ-R was developed using an established translation process (forward and back translation), then tested for validity and reliability in the current study. The scale was translated from English to Thai, then translated from Thai back to English by two independent experienced translators. The Thai translation was then reviewed by two faculty medical instructors (from the Department of Pediatrics and the Department of Psychiatry). On the basis of the instructors’ suggestions, minor cultural adaptation was necessary to suit the Thai context while maintaining the quality of the instrument. Next, three raters (a native English speaker, and two experts in medical education) rated the degree of agreement between the original and back-translated versions. Another three experts in medical education evaluated the content validity and congruency using a rating scale from 1 to 4. Finally, a pilot study was performed among 30 medical students to check for any difficulties in understanding the content and completing the questionnaire.

Following minor cultural adaptation to suit the Thai context, the Thai version of the Clinical Workplace Learning NAQ-R revealed satisfactory internal consistency (Cronbach’s alpha: 0.922) and a good degree of interrater agreement with the original version (83.9%). The S-CVI and ACP were both 0.91. Therefore, this instrument was considered to be acceptable for data collection.

#### 3) Student-related factors

The third part of the survey consisted of self-assessment, covering students’ experiences of related factors to mistreatment such as burnout, risk for depression, unprofessional behaviors, and motivation.

A Thai version of the Maslach Burnout Inventory–Student Survey was previously developed and validated, with an acceptable Kappa value of 0.83, and good internal consistency (Cronbach’s alpha: 0.80). The Cronbach’s alpha coefficient values for emotional exhaustion, depersonalization, and personal accomplishment were 0.89, 0.81, and 0.70 respectively [35].

The risk of depression was identified using the Thai version of the Patient Health Questionnaire (PHQ-9). This questionnaire had satisfactory internal consistency (Cronbach’s alpha: 0.79) and moderate convergent validity with the Hamilton Rating Scale for Depression (*r* = 0.56; *p* < 0.001). The categorical algorithm of the PHQ-9 had low sensitivity (0.53) but very high specificity (0.98) and a high positive likelihood ratio (27.37). Used as a continuous measure, the optimal PHQ-9 cut-off score of 9 showed sensitivity of 0.84, specificity of 0.77, a positive predictive value of 0.21, a negative predictive value of 0.99, and a positive likelihood ratio of 3.71. The area under the curve was 0.89 (standard deviation [SD]: 0.05, 95% CI: 0.85 to 0.92) [36].

### Statistical analysis

Descriptive statistics were used to present participants’ demographic data, mistreatment reporting actions, the prevalence of mistreatment, the categories and the person conducting the mistreatment, and student-related factors (burnout, risk of depression, unprofessional behavior, and motivation).

For the Thai version of the Clinical Workplace Learning NAQ-R, interrater reliability (Kappa) was used to identify the extent of agreement. Internal consistency was analyzed to demonstrate the level of reliability and measured with a standardized Cronbach’s alpha coefficient. A Cronbach’s alpha > 0.8 was considered acceptable. Content validity and congruency were summarized using the content validity index for scale (S-CVI) and average congruency percentage (ACP). S-CVI was calculated by summing the content validity for each item, which was measured from the ratio of experts who rated a score of 3 or 4 for each item, divided by the total items. The ACP was calculated by summing the ratio of items that each expert rated as 3 or 4 divided by number of the total experts. S-CVI and ACP values > 0.9 were considered acceptable.

Multivariate analysis of variance was used to determine the associations between experiences of mistreatment and each factor. Associations between unprofessionalism and mistreatment were measured and displayed as crude odds ratios (ORs). All results were discussed with all collaborators.

This study was reviewed and approved by the Human Research Ethics Committee, Faculty of Medicine Ramathibodi Hospital, Mahidol University, Thailand (protocol number MURA2020/1768).

## Results

The response rate was 79.1%. The full panel of questionnaires was completed by 56.0% of the participants (*N* = 681, 52.4% female) that agreed to participate, and were included in the analysis. Over half (54.6%) of the participants were clinical medical students (years 4–6). The results of the Thai version of the Clinical Workplace Learning NAQ-R indicated that 74.5% of participants (*n* = 510) had been mistreated at least once in the past year, of which 67.3% were medical students in the clinical phase.

### Demographic data

31.4% self-reported at least one underlying disease (e.g., allergic rhinitis, atopic dermatitis, asthma, and seborrheic dermatitis). In addition, 8.2% self-reported at least one psychiatric problem (e.g., depression, attention deficit hyperactivity disorder, anxiety disorder). Age, gender, GPA, smoking and alcohol consumption are shown in Table 1.

**Table 1:** Participants’ demographic characteristics (*N* = 681)

Baseline characteristics	Total		Clinical medical students (years 4–6)	
	***n* = 681**	**(%)**	***n* = 372**	**(%)**
**Age, years**				
< 19	120	(17.7)	-	-
20–21	202	(29.7)	30	(8.1)
22–23	226	(33.2)	214	(57.7)
24–25	124	(18.2)	122	(32.9)
≥ 26	8	(1.2)	5	(1.4)
**Gender**				
Male	320	(47.0)	182	(48.9)
Female	357	(52.4)	187	(50.3)
Non-specified	4	(0.6)	3	(0.8)
**Current year in medical school**				
Year 1	88/204 (43.1%)	(12.9)	-	-
Year 2	92/211 (43.6%)	(13.5)	-	-
Year 3	129/211(61.1%)	(18.9)	-	-
Year 4	121/222 (54.5%)	(17.8)	121/222 (54.5%)	(32.5)
Year 5	117/198 (59.1%)	(17.2)	117/198 (59.1%)	(31.5)
Year 6	134/169 (79.3%)	(19.7)	134/169 (79.3%)	(36.0)
**GPA**				
< 2.50	12		9	(2.4)
2.50–2.79	30	(4.5)	26	(7.1)
2.80–2.99	58	(8.7)	40	(10.8)
3.00–3.24	93	(14.0)	72	(19.5)
3.25–3.59	196	(29.5)	131	(35.5)
≥ 3.60	276	(41.5)	91	(24.7)
**Smoking**				
Yes	12	(1.8)	11	(3.0)
No	665	(98.2)	361	(97.0)
**Alcohol consumption**				
Yes	174	(25.7)	105	(28.3)
No	503	(74.3)	266	(71.7)
**Underlying disease(s)**				
Yes	214	(31.4)	152	(40.9)
No	467	(68.6)	220	(59.1)
**Psychiatric disorder(s)**				
Yes	56	(8.2)	33	(8.9)
No	625	(91.8)	339	(91.1)

*GPA* Grade point average

**Fig. 1:**
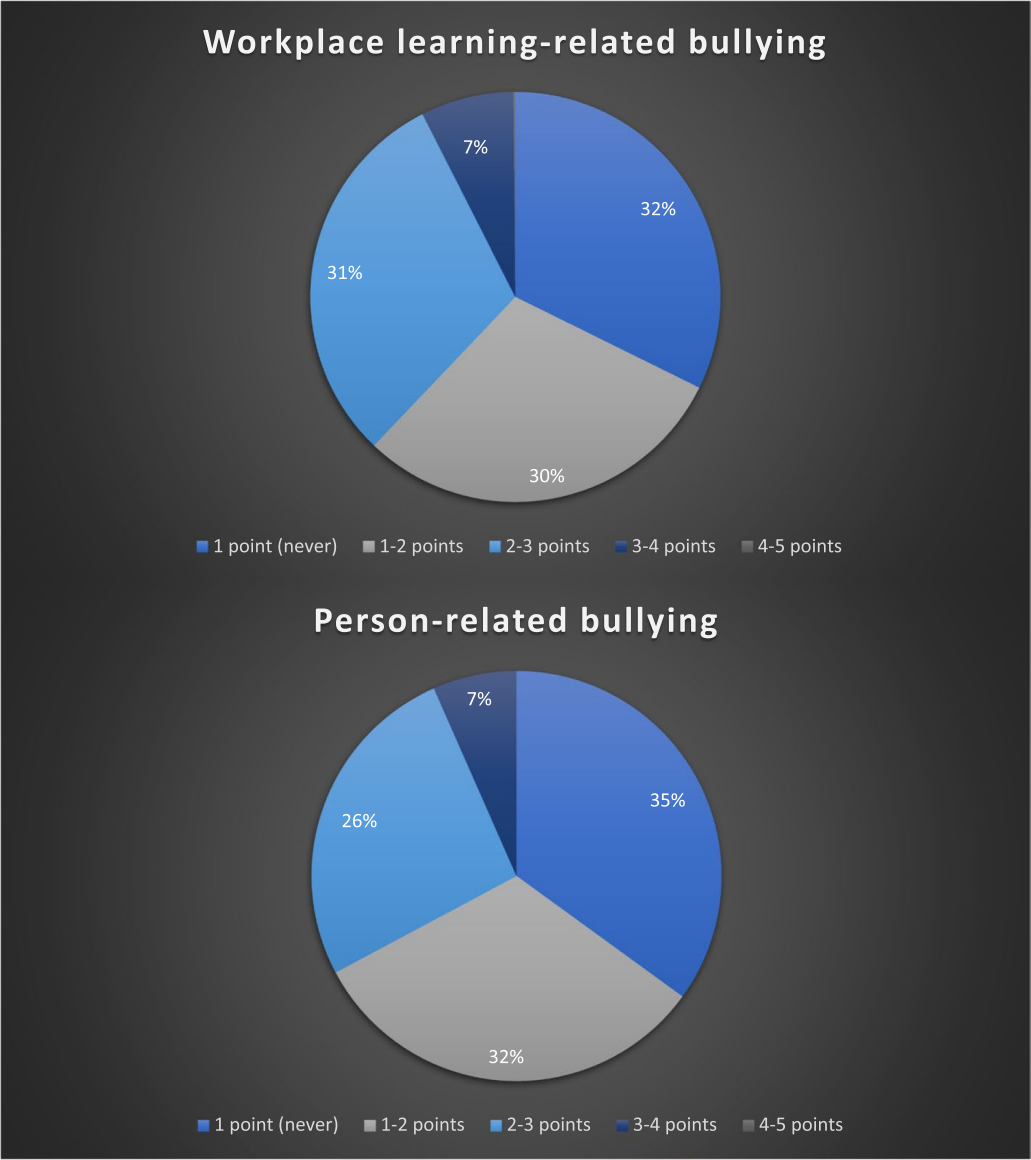
Frequency of workplace learning-related bullying and person-related bullying as rated by medical students

Workplace learning-related bullying was the most commonly reported mistreatment category (67.7%, Table 2), followed by person-related bullying (65%). Detailed results are shown in Fig. 1.

The mistreatment results revealed that students in the clinical phase had higher mean (SD) scores for the frequency of mistreatment compared with the mean score in all years (Table 2). Attending staff or teachers were the primary source of mistreatment experienced by medical students (31.6%) (Table 3). The most common source of mistreatment among preclinical medical students was senior students or peers (25.9%) whereas the most common source among clinical students was attending staff (57.5%). The most common way that participants chose to respond when being mistreated was talking with peers or friends (49.6%). Most students (85.3%) believed that the personal character of the person conducting the mistreatment was the cause of the mistreatment of their medical student peers.

**Table 2:** Results for mistreatment based on the Thai version of the Clinical Workplace Learning Negative Acts Questionnaire-Revised

Categories of mistreatment	Total (*N* = 681)			Clinical medical students (*n* = 372)		
	**Mean (SD) (range 1–5)**		**Mistreated n (%)**	**Mean (SD) (range 1–5)**		**Mistreated n (%)**
Workplace learning-related bullying	1.70	(0.75)	461 (67.7)	2.06	(0.76)	320 (86.0)
Person-related bullying	1.82	(0.79)	443 (65.1)	2.18	(0.78)	319 (85.8)
Physically intimidating bullying	1.49	(0.69)	283 (41.6)	1.78	(0.75)	245 (65.9)
Sexual harassment	1.29	(0.60)	180 (26.4)	1.46	(0.73)	149 (40.1)
Ethnic harassment	1.14	(0.47)	80 (11.8)	1.22	(0.59)	68 (18.3)

**Table 3:** Other results related to mistreatment, participants’ opinion about the mistreatment situation among faculty, consequences (burnout, depression, unprofessionalism, and motivation), and mistreatment reports

Mistreatment	Total		Clinical medical students	
	***N* = 681**	**(%)**	***n* = 372**	**(%)**
**Source of mistreatment**				
Attending staff	215	(31.6)	214	(57.5)
Registered nurse(s) or practical nurse(s)	196	(28.8)	191	(51.3)
Resident(s), fellows or other physician(s)	175	(25.7)	173	(46.5)
Senior student(s) or peer(s)	166	(24.4)	86	(23.1)
Lecturer(s)	119	(17.5)	52	(14.0)
Department officer(s)	87	(12.8)	78	(21.0)
Faculty officer(s)	43	(6.3)	36	(9.7)
Patient(s)	41	(6.0)	40	(10.8)
**Participants’ responses chose being mistreated**				
Talk with peers or friends	338	(49.6)	248	(66.7)
Ignore the situation	175	(25.7)	128	(34.4)
Talk to family members	149	(21.9)	110	(29.6)
Talk to senior students	45	(6.6)	31	(8.3)
Inform professors of the situation	42	(6.2)	34	(9.1)
Talk to someone mistreating directly	29	(4.3)	17	(4.6)
Consult therapists	17	(2.5)	14	(3.8)
**Participants’ opinion about the mistreatment event among faculty**				
Personal character of the mistreating person	581	(85.3)	326	(87.6)
Work-related stress	398	(58.4)	221	(59.4)
Strict hierarchical system	338	(49.6)	180	(48.4)
Organizational culture that accepts mistreatment	296	(43.5)	157	(42.2)
Lack of mistreatment management policy	283	(41.6)	145	(39.0)
**Consequences**				
**Burnout**	236	(34.7)	142	(38.2)
**Risk for depression (PHQ-9)**				
No	62	(9.1)	40	(10.8)
Mild risk	199	(29.2)	109	(29.3)
Moderate risk	191	(28.1)	95	(25.5)
Severe risk	181	(26.6)	99	(26.6)
Very severe risk	48	(7.1)	29	(7.8)
**Unprofessionalism**				
Go to work or class late without reasonable cause	205	(30.1)	97	(26.1)
Do not submit work or submit work late	182	(26.7)	113	(30.4)
Do not care or take responsibility in patient care	129	(18.9)	121	(32.5)
Have conflict with peers	121	(17.8)	89	(23.9)
Absent from work or class without reasonable excuse	99	(14.5)	23	(6.2)
Enter untrue information in the medical record or patient report	73	(10.7)	67	(18.0)
Show mistreatment behaviors to another people	71	(10.4)	42	(11.3)
Reported a case untruthfully	35	(5.1)	29	(7.8)
**Mistreatment report**	56	(8.2)	50	(13.4)
**Report to participants’ department (*n* = 56)**				
Other professor(s)	24	(42.9)	20	(35.7)
Course coordinator	18	(32.1)	17	(30.4)
Medical student council or club	5	(8.9)	4	(7.1)
Department officer(s)	4	(7.1)	4	(7.1)
Student affairs	3	(5.4)	2	(3.6)
Medical education section	3	(5.4)	3	(5.4)
**Satisfaction with reporting the situation (*n* = 54)**				
Very satisfied	3	(5.6)	2	(3.7)
Satisfied	8	(14.8)	8	(14.8)
Neutral	22	(40.7)	18	(33.3)
Dissatisfied	14	(25.9)	13	(24.1)
Very dissatisfied	7	(13.0)	7	(13.0)

### Unprofessional behavior and well-being of medical students

Almost one-third (30.1%) of participants self-reported unprofessional behaviors such as going to work or class late without reasonable cause. In the Clinical when rotating through the wards phase, the self-reported unprofessional behavior most often mentioned were students’ lack of self-care or lack of taking responsibility in patient care.

Overall, 34.7% of participants reported burnout, with a higher percentage of burnout among students in the clinical phase rotations compared with medical students in the pre-clinical phase years (38.2% vs. 30.4%). In addition, 7.1% of participants exhibited severe depression on the PHQ-9 assessment, with a higher percentage of medical students in the clinical phase years exhibiting depression compared with preclinical medical students (7.8% vs. 6.2%).

### Mistreatment reporting actions

Only 8.2% of medical students had formally reported a mistreatment event to another person (Table 3). The primary recipients of medical students’ reports regarding these events were faculty members (42.9%) and course directors (32.1%). However, only 20% of those who had ever reported an event, reported that they were “satisfied or very satisfied” with the reporting process.

### Association between mistreatment of clinical medical students and medical student-related factors

Age, gender, current year of study, GPA, smoking, alcohol consumption, risk of depression, motivation, and burnout were explored for potential correlations with mistreatment of medical students in the clinical phase using a full model analysis (Table 4). The results regarding student-related factors showed that the year of study had a weak positive significant association with workplace-related bullying (*r* = 0.26, *p* < 0.001), person-related bullying (*r* = 0.22, *p* < 0.001), physically intimidating bullying (*r* = 0.19, *p* < 0.001), and sexual harassment (*r* = 0.16, *p* < 0.001), and a modest significant association with ethnic harassment (*r* = 0.08, *p* = 0.038). This suggested that students in different years experienced different types and frequencies of mistreatment (Table 2).

**Table 4:** Factors related to mistreatment among clinical medical students (*n* = 372)

Factors (*n* = 372)	Workplace learning-related bullying			Person-related bullying			Physically intimidating bullying			Sexual harassment			Ethnic harassment		
	**Coef.**	**(SE)**	***p***	**Coef**	**(SE)**	***p***	**Coef.**	**(SE)**	***p***	**Coef.**	**(SE)**	***p***	**Coef.**	**(SE)**	***p***
Current year	0.26	(0.06)	< 0.001***	0.22	(0.05)	< 0.001***	0.19	(0.05)	< 0.001***	0.16	(0.05)	< 0.001***	0.08	(0.04)	0.038*
Risk for depression (PHQ-9)	0.17	(0.04)	< 0.001***	0.20	(0.03)	< 0.001***	0.13	(0.03)	< 0.001***	0.11	(0.03)	0.001**	0.07	(0.03)	0.015*
Age	− 0.06	(0.04)	0.103	0.07	(0.03)	0.023*	0.06	(0.03)	0.038*	0.08	(0.03)	0.005**	0.05	(0.02)	0.049*
Sex (male)	0.17	(0.08)	0.036*	0.11	(0.08)	0.155	0.01	(0.08)	0.950	− 0.20	(0.08)	0.009**	− 0.06	(0.06)	0.331
Motivation	− 0.07	(0.06)	0.186	− 0.17	(0.05)	0.001**	− 0.10	(0.05)	0.044*	0.02	(0.05)	0.745	− 0.04	(0.04)	0.354
Burnout	0.05	(0.08)	0.558	0.20	(0.08)	0.012*	0.13	(0.08)	0.095	0.10	(0.08)	0.195	0.09	(0.06)	0.176
Alcohol consumption	0.02	(0.09)	0.786	0.08	(0.09)	0.342	− 0.08	(0.09)	0.342	0.20	(0.08)	0.015*	0.07	(0.07)	0.311
GPA	0.16	(0.11)	0.165	0.01	(0.12)	0.903	0.13	(0.11)	0.233	0.05	(0.11)	0.669	− 0.05	(0.09)	0.600
Smoking	− 0.10	(0.25)	0.676	− 0.32	(0.23)	0.175	< 0.01	(0.23)	0.986	0.08	(0.22)	0.708	− 0.06	(0.18)	0.732

*GPA* Grade point average, *PHQ,* Patient Health Questionnaire, *SE* Standard error

^***^ statistically significant at *p* < 0.001, ** statistically significant at *p* < 0.01, * statistically significant at *p* < 0.05

The risk of depression had modest but significant associations with workplace-related bullying (*r* = 0.17, *p* < 0.001), person-related bullying (*r* = 0.20, *p* < 0.001), and physically intimidating bullying (*r* = 0.13, *p* < 0.001), a moderate significant association with sexual harassment (*r* = 0.11, *p* = 0.001), and a modest significant association with ethnic harassment (*r* = 0.07, *p* = 0.015). Therefore, mistreatment was related to the risk of depression.

Age showed a moderate significant association with sexual harassment (*r* = 0.08, *p* = 0.005), and modest significant associations with person-related bullying (*r* = 0.07, *p* = 0.023), physically intimidating bullying (*r* = 0.06, *p* = 0.038), and ethnic harassment (*r* = 0.05, *p* = 0.049). Students of different ages faced different types and frequencies of mistreatment. Gender (male) had a moderate significant association with sexual harassment (*r* = 0.20, *p* = 0.009), and a modest significant association with workplace-related bullying (*r* = 0.17, *p* = 0.036). Motivation had a moderate significant association with person-related bullying (*r* = 0.17, *p* = 0.001) and a modest significant association with physically intimidating bullying (*r* = 0.10, *p* = 0.044). Therefore, lack of motivation had negative impacts in some mistreatment categories.

Alcohol consumption had a modest significant association with sexual harassment (*r* = 0.20, *p* = 0.015), suggesting that students who had a history of drinking alcohol were more likely to experience sexual harassment. Burnout had a modest significant association with person-related bullying (*r* = 0.20, *p* = 0.012). GPA and smoking did not have significant associations with any mistreatment category.

### Correlations between mistreatment and unprofessionalism among medical students in the clinical phase

Features of unprofessional behavior were correlated with mistreatment, such as reporting a patient’s case untruthfully, having conflicts or arguments with colleagues, absence from class or work without acceptable cause, reasons beyond the norm and mistreating others (Table 5).

**Table 5:** Correlations between unprofessionalism and clinical medical students’ mistreatment (*n* = 372)

Unprofessionalism (*n* = 372)	Workplace learning-related bullying		Person-related bullying		Physically intimidating bullying		Sexual harassment		Ethnic harassment	
	**Crude OR**	***p***	**Crude OR**	***p***	**Crude OR**	***p***	**Crude OR**	***p***	**Crude OR**	***p***
Absent from class or work without reasonable cause	1.44	0.191	1.91	0.021^*^	1.40	0.217	1.01	0.973	0.60	0.368
Attend class or work late without reasonable cause	1.10	0.529	1.21	0.224	1.22	0.209	1.09	0.587	0.98	0.910
Faked medical records or patient reports	1.20	0.298	1.04	0.821	1.08	0.655	1.31	0.110	1.09	0.687
Reported patient’s case untruthfully	1.81	0.020^*^	1.51	0.099	1.29	0.310	2.36	< 0.001***	1.59	0.047^*^
Submitted work late	0.84	0.243	1.02	0.916	0.89	0.456	0.86	0.362	0.70	0.121
Had conflicts with colleagues	1.52	0.009^**^	1.63	0.002^**^	1.16	0.366	1.30	0.095	1.12	0.548
Performed mistreatment behaviors	1.40	0.111	1.54	0.043^*^	1.25	0.299	1.35	0.135	1.24	0.371
Inadequate patient care	1.19	0.222	1.15	0.351	0.94	0.670	1.08	0.627	0.92	0.663

*OR* Odds ratio

^***^ statistically significant at *p* < 0.001, ** statistically significant at *p* < 0.01, * statistically significant at < 0.05

## Discussion

The current results revealed that the Thai Clinical Workplace Learning NAQ-R has a high level of reliability, with a Cronbach’s alpha value of 0.922, and a high degree of agreement with the original version of the scale (83.9%). The proportion of medical students reporting mistreatment experiences was 74.5%. The most common type of reported mistreatment was workplace learning-related bullying (67.7%) and the most common source was attending staff (31.6%). However, the most common sources of mistreatment for preclinical medical students were senior students or peers (25.9%), whereas clinical students most often reported mistreatment by attending staff (57.5%). Surprisingly, only 8.2% of medical students who were mistreated, formally reported these mistreatment events to others. The academic year was significantly related to mistreatment, with a higher incidence of mistreatment in the clinical phase learning environment. Risk of depression and burnout were significantly associated with mistreatment categories, such as person-related bullying (depression: *r* = 0.20, *p* < 0.001, burnout: *r* = 0.20, *p* = 0.012). Experiences of mistreatment among students had a significant relationship with reports of unprofessional behavior, such as having conflicts or arguments with colleagues, absence from class or work without reasonable cause, and mistreatment of others.

### Medical students’ experiences of mistreatment

The prevalence of medical students’ experiences of mistreatment in medical school in the current study was 74.5%, which is comparable to that reported in the United States (63.9% by faculty members and 75.5% by residents) and Japan (68.5%) [7, 10, 17]. Despite the Safe Medical School Campaign launched in Thailand in 2021 to raise awareness of the mistreatment problem, the prevalence of mistreatment in the current study was higher than the previous study in 2019 (63.7%) [11]. Therefore, the effectiveness of the Safe Medical School Campaign should be evaluated. The reasons for the difference in prevalence could stem from the different context and learning environment between institutions and the different research tools (Mistreatment perception versus the Thai version of the Clinical Workplace Learning NAQ-R) used for measurement. Workplace learning-related bullying was one of the most common types of mistreatments, consistent with previous studies. Mistreatment and disrespectful behaviors are reported to be related to the culture in the medical school and characteristics of the clinical workplace, such as hierarchy, medical culture, pressure on medical students and stressful health care environments [29, 31].

The primary source of mistreatment in the current study was attending staff who directly supervised and taught medical students (31.6%). This was in contrast to previous studies in the United States (63.9%) and Japan (45.2%) that reported faculty members as the primary source of mistreatment [7, 10, 30]. However, when only pre-clinical year students were explored, the results revealed that senior students and peers were the main source of mistreatment (25.9% in pre-clinical year students). This may be because pre-clinical students spend less time in the clinical environment and interact more with senior students in both intra- and extra-curricular activities compared with clinical year students. These results suggest that faculty development programs are needed to create safer learning environments, especially in clinical settings. Not only faculty members, but also residents, fellows, student peers, other health professionals, and educational staff need to be included in such programs to promote a safer learning environment to support medical students.

### Mistreatment reporting actions

Only 8.2% of students in the current study had filed an official report of mistreatment to someone on the faculty. This proportion was low compared with the 30% report rate observed in previous studies [4]. Possible reasons for not reporting a mistreatment event include fear of reprisal, time- and energy-consuming reporting processes, and the incident being of insufficient severity to report [30, 31]. This phenomenon may be similar to the “failure to fail” phenomenon, in which a person is reluctant to report a negative result and prefers to remain silent about unpleasant messages. The reluctance to report negative events may also be related to fear about harming one’s reputation, and uncertainty about the consequences of reporting [37]. Students’ decisions about whether to report mistreatment may be influenced by the cost of the potential outcome, such as their ability to trust and be safe within the institution [38]. Therefore, a suitable reporting system should consider what the consequences are likely to be for those who report mistreatment and those who are reported. Future investigations should explore the reasons and factors underlying this underreporting. An increase in the number and nature of mistreatment reports by mistreated students and bystanders may be helpful for developing an effective reporting system for identifying problems and finding solutions.

### Association between mistreatment of medical students in the clinical phase and student-related factors

The current year of study had significant associations with workplace-related bullying, person-related bullying, physically intimidating bullying, and sexual harassment. This was the most significant factor associated with mistreatment among clinical medical students, and was consistent with the findings of a previous study in medical students in Southern Thailand [11]. It can be hypothesized that students in the higher clinical years’ experience more unsafe clinical learning environments or more intense hierarchical cultures. Further studies should focus on the potential causes of mistreatment in different clinical years. The risks of depression and burnout were significantly associated with mistreatment categories. This finding was in alignment with those of previous studies, confirming that mistreatment is associated with medical students’well-being in general in both Western and Asian context [7, 14, 17–19].

### Correlations between mistreatment and reported lack of professionalism of medical students in the clinical year level

Reporting a patient’s case untruthfully was the most common type of unprofessional behavior self-reported by those that were also mistreated, as well as having conflicts or arguments with colleagues, being absent from class or work without reasonable cause, and mistreatment of others. Reporting a patient’s case untruthfully, having conflicts or arguments with colleagues, and mistreatment of others are dishonest and disrespectful behaviors that were mentioned as students’ unprofessional behavior among both Asian and Western students [39]. Previous studies observed that experiencing mistreatment is related to burnout, which is in turn associated with sub-optimal patient care and attrition from the medical profession [40–42]. In this study, mistreatment and self-reported unprofessional behavior of medical students were correlated. It has been reported that some individuals may accept and enculturate mistreatment into their future practice [43]. A previous study in Thailand reported the effects of poor mental health and burnout in Thai medical students, which caused a reduction in empathy [44, 45]. Therefore, it may refer to these as possible effects of mistreatment.

### Strengths and limitations

This study revealed evidence of mistreatment among medical students, associations between types of mistreatment and student-related factors, the reporting behavior and related well-being of medical students. The finding may be beneficial for medical schools in designing novel approaches for detecting and controlling mistreatment and supporting mistreated students.

However, this study examined students at one institution and may not be representative of the general population of Asian medical students. Additionally, the findings in this study were based on student self-reporting which may not be compatible with other perspectives. Moreover, this correlational study did not enable us to draw conclusions regarding the direction of causality. Further studies will be needed to explore the cause and effect of the phenomena observed in the current study.

Additionally, there might be potential confounding factors that influenced the mistreatment report, such as differences in understanding and interpretation of the mistreatment concept, students' values and beliefs, and personality traits. Therefore, this study may not impartially represent the mistreatment situation of *all* Thai medical students. A future multi-center qualitative study is recommended to explore students' perceptions of mistreatment more in depth in greater depth.

## Conclusions

Mistreatment of medical students is a serious problem worldwide, including in Thailand. A high prevalence of mistreatment was revealed, and workplace learning-related bullying was the most common category. Attending staff were the most common source of mistreatment. Mistreatment was associated with several negative consequences, including burnout, risk of depression, and unprofessionalism. However, the rate of mistreatment reporting by medical students was low. Therefore, increasing awareness among medical students and related healthcare providers is necessary. In addition, faculty development programs should address this issue and a systematic mistreatment reporting system should be developed to capture mistreatment in medical schools.

## Data Availability

The datasets generated and/or analysed during the current study are not publicly available due sensitive confidential issues but are available from the corresponding author on reasonable request.

## Abbreviations

AAMC: The Association of American Medical Colleges
ACP: Average congruency percentage
AMA: The American Medical Association
EH: Ethnic mistreatment
GPA: Grade point average
NAQ-R: The Negative Acts Questionnaire–Revised
ORs: Crude odds ratios
PHQ-9: The Patient Health Questionnaire (PHQ-9)
PIB: Physically intimidating bullying
PRB: Person-related bullying
S-CVI: The content validity index for scale
SH: Sexual harassment
WLRB: Workplace learning-related bullying
